# Regulation of diabetic cardiomyopathy by caloric restriction is mediated by intracellular signaling pathways involving ‘SIRT1 and PGC-1α’

**DOI:** 10.1186/s12933-018-0754-4

**Published:** 2018-08-02

**Authors:** Maayan Waldman, Keren Cohen, Dor Yadin, Vadim Nudelman, Dan Gorfil, Michal Laniado-Schwartzman, Ran Kornwoski, Dan Aravot, Nader G. Abraham, Michael Arad, Edith Hochhauser

**Affiliations:** 1 Cardiac Research Laboratory, Felsenstein Medical Research Institute Petah-Tikva, Sackler Faculty of Medicine, Tel Aviv University, Tel Aviv, Israel; 2 Leviev Heart Center, Sheba Medical Center, Tel Hashomer and Sackler School of Medicine, Tel Aviv University, Tel Aviv, Israel; 3 Department of Pharmacology, New York Medical College, Valhalla, NY 10595 USA; 4 Felsenstein Research Center, Rabin Medical Center, Sackler Faculty of Medicine, Tel Aviv University, Jabotinsky St, 49100 Petach Tikva, Israel

**Keywords:** Caloric restriction, Cardiomyopathy, Diabetes mellitus, SIRT1, PGC-1α

## Abstract

**Background:**

Metabolic disorders such as obesity, insulin resistance and type 2 diabetes mellitus (DM2) are all linked to diabetic cardiomyopathy that lead to heart failure. Cardiomyopathy is initially characterized by cardiomyocyte hypertrophy, followed by mitochondrial dysfunction and fibrosis, both of which are aggravated by angiotensin. Caloric restriction (CR) is cardioprotective in animal models of heart disease through its catabolic activity and activation of the expression of adaptive genes. We hypothesized that in the diabetic heart; this effect involves antioxidant defenses and is mediated by SIRT1 and the transcriptional coactivator PGC-1α (Peroxisome proliferator-activated receptor-γ coactivator).

**Methods:**

Obese Leptin resistant (*db/db*) mice characterized by DM2 were treated with angiotensin II (AT) for 4 weeks to enhance the development of cardiomyopathy. Mice were concomitantly either on a CR diet or fed ad libitum. Cardiomyocytes were exposed to high levels of glucose and were treated with EX-527 (SIRT1 inhibitor). Cardiac structure and function, gene and protein expression and oxidative stress parameters were analyzed.

**Results:**

AT treated *db/db* mice developed cardiomyopathy manifested by elevated levels of serum glucose, cholesterol and cardiac hypertrophy. Leukocyte infiltration, fibrosis and an increase in an inflammatory marker (TNFα) and natriuretic peptides (ANP, BNP) gene expression were also observed. Oxidative stress was manifested by low SOD and PGC-1α levels and an increase in ROS and MDA. DM2 resulted in ERK1/2 activation. CR attenuated all these deleterious perturbations and prevented the development of cardiomyopathy. ERK1/2 phosphorylation was reduced in CR mice (p = 0.008). Concomitantly CR prevented the reduction in SIRT activity and PGC-1α (p < 0.04). Inhibition of SIRT1 activity in cardiomyocytes led to a marked reduction in both SIRT1 and PGC-1α. ROS levels were significantly (p < 0.03) increased by glucose and SIRT1 inhibition.

**Conclusion:**

In the current study we present evidence of the cardioprotective effects of CR operating through SIRT1 and PGC-1 α, thereby decreasing oxidative stress, fibrosis and inflammation. Our results suggest that increasing SIRT1 and PGC-1α levels offer new therapeutic approaches for the protection of the diabetic heart.

## Introduction

Diabetes mellitus type 2 (DM2) is a common metabolic disorder characterized by impaired glucose tolerance and is associated with excess cardiovascular morbidity and mortality [1, 2]. Diabetic cardiomyopathy is manifested by increased cardiomyocyte stiffness and fibrotic changes. Progressive cardiac fibrosis found in diabetic cardiomyopathy and during pressure overload results in diastolic dysfunction leading to reduced myocardial contractility, pulmonary congestion and, ultimately, heart failure [3, 4]. Despite its clinical significance, the pathophysiologic basis of myocyte hypertrophy and stiffening as well as cardiac fibrosis in DM2 remains poorly understood [5, 6]. The primary factors that lead to cardiomyocyte injury and dysfunction in diabetes comprise insulin resistance and hyperinsulinemia, hyperglycemia, and elevated free fatty acids (FFA) leading to myocardial lipotoxicity [7]. Secondary mediators include oxidative stress, mitochondrial dysfunction, abnormal intracellular calcium metabolism [8] and chronic inflammation [9]. Angiotensin II (AT), the effector peptide of the renin-angiotensin system (RAS), is a potent vasoconstrictor [10]. Cardiac AT synthesis is sufficient to trigger the development of cardiac hypertrophy [11]. While hypertensive doses of AT produce a more pronounced hypertrophic response, angiotensin receptor type 1 (AT1R)-mediated signaling may induce cardiac hypertrophy independently of hypertension [12] by acting as a cardiac growth factor and through induction of the “fetal” gene TGF-β as well as dysregulation of collagen degrading matrix metalloproteinases (MMPs) [13, 14]. AT upregulation exacerbates the cardiac phenotype of diabetic mice [15].

Caloric restriction (CR) is defined as a decrease in caloric intake without deprivation of essential nutrients. Observational studies of individuals voluntarily practicing long-term CR suggest that it positively affects cardiovascular disease risk factors [16, 17]. CR increases longevity and both delays and slows the progression of multiple age-related diseases in animal models of cardiac disease [18, 19]. Adiponectin, which increased in the plasma after CR [20, 21], has been implicated in CR-induced cardioprotection [20]. SIRT1, a redox-sensitive enzyme, is a member of a large family of class III histone deacetylase (HDAC) [22, 23]. SIRT1 is a modulator of genetic stability by extending life span in yeast, flies, and worms, while its deletion shortens life span [24]. Concomitantly, SIRT1 regulates a wide variety of cellular processes including apoptosis/cell survival, endocrine signaling, chromatin remodeling, and gene transcription [25]. SIRT1 activation recapitulates many of the molecular events downstream of CR in vivo, including enhancing mitochondrial biogenesis through activation of Peroxisome proliferator-activated receptor gamma coactivator 1-alpha (PGC-1α), improving metabolic signaling pathways, and blunting pro-inflammatory pathways [26, 27]. This effect is partly mediated by an anti-oxidant protein superoxide dismutase (SOD) [28, 29]. We have previously reported that CR decreased inflammatory markers in the diabetic heart [30].

DM2 often coexists with hypertension and is associated with increased AT signaling. We developed a murine cardiomyopathy mouse model by further stressing the diabetic heart by AT [30], and hypothesized that CR will affect cardiac remodeling in these hearts through molecular mechanisms related to anti-oxidative pathways. We identified SIRT1, and PGC-1α as the driving force behind the cardioprotective effect of CR.

## Methods

### Animal model

The animal experiments were approved by the Institutional Animal Care and Use Committee of Tel Aviv University (M-15-010). Homozygous *db/db* mice (C57BLKS/J-*leprdb/leprdb*) and their wild type (WT) littermates were maintained in a pathogen-free facility on regular rodent chow with free access to water and 12-h light and dark cycles. Homozygous mice were verified by PCR. Male WT or *db/db* mice (12–14 weeks old) were used for the experiments. *db/db* mice develop mild cardiomyopathy at an advanced age [2, 31]. To enhance development of heart disease and obtain a robust phenotype, mice were stressed by ATII as described in other cardiomyopathy models [12]. The AT administration is described below (“Angiotensin” section). Mice were divided into the following groups (n = 5–14 each in each group): WT (n = 10), WT + AT (n = 12), WT + AT + CR (n = 5), *db/db (n *= *14), db/db *+ AT(n = 14), *db/db *+ AT + CR (n = 8).

### Echocardiography

Animals were lightly anesthetized by inhaling isoflurane. The heart rate in all animals was maintained during the recording at 450–500 b/m. Two-dimensional (2D) guided M-mode echocardiography was performed using an echocardiogram (Vevo 2100 Imaging System, VisualSonics, Toronto, Ontario, Canada) equipped with a 30-MHz linear transducer. The 2D mode in the parasternal long-axis view was used to monitor the heart. From this view, an M-mode cursor was positioned perpendicular to the interventricular septum and posterior wall of the left ventricle (LV) at the level of the papillary muscles. An M-mode image was obtained at a sweep speed of 100 mm/s. Left-ventricular end-diastolic dimensions (LVEDD) and left-ventricular end-systolic chamber dimensions (LVESD) were measured. The percentage of left-ventricular fractional shortening (FS) was calculated as [(LVEDD—LVESD)/LVEDD] × 100 [32, 33].

### Angiotensin

Mice were anesthetized with 2% isoflurane and an ALZET osmotic pump (Durect Corp., Cupertino, CA, USA) was subcutaneously implanted into each mouse. The osmotic pumps infused angiotensin II (Sigma-Aldrich, St. Louis, MO, USA) at a rate of 1000 ng kg−^1^ min^−1^ for 4 weeks.

### Blood pressure measurement

Systolic blood pressure (SBP) was measured at the end of the experiment in awake mice using a noninvasive computerized tail-cuff system (Blood pressure pump, Life science instrument, CA, USA). Mice were placed in temperature-controlled chambers (37 °C) and blood pressures were recorded in 2–3 cycles of 10 measurements.

### Biochemical measurement in the serum

Serum (200 µl) was collected and kept on ice until processed. Glucose, cholesterol, triglycerides, aspartate transaminase (AST) and alanine transaminase (ALT) levels were determined in duplicate, using a commercial Olympus OSR6126 kit (Center Valley, PA, USA) according to the manufacturer’s protocols [33].

### Caloric restriction

Mice were housed in individual cages. Caloric restricted (CR) mice were fed 90% of their average caloric intake for 2 weeks (10% restriction), followed by 65% of that for an additional 2 weeks (35% restriction). Experiments were conducted after the 4-week period as we have previously published [32].

### Histopathology

Midventricular heart sections were fixed in 4% formalin, and then embedded in paraffin. Several transverse sections were cut from the paraffin-embedded samples and stained with Hematoxylin and Eosin. Sections from each heart were also stained with Masson trichrome for collagen deposition [33, 34]. For each staining, slides from 3 mice per group were analyzed.

### Western blotting

Cardiac tissue (n = 4 in each group) was homogenized in lysis buffer and quantified for protein levels using a commercial assay (Bio-Rad, CA, USA). Western blotting was performed according to standard procedures as previously described [33]. Protein samples (60 μg) were applied to sodium dodecyl sulfate (SDS) polyacrylamide gel (10–15%), electrophoresed under denaturing conditions and electrotransferred onto nitrocellulose membranes (Bio-Rad). Membranes were blocked with 3% BSA in Tris-buffer saline (TBS). Primary antibodies for phosphorylated and total ERK1/2, PPARγ, β ACTIN, GAPDH (Santa Cruz Biotechnology, Dallas, Texas, USA), PGC-1α (ABCAM, Cambridge, UK), SOD2 (ABCAM, Cambridge, UK), and SIRT1 (Merck Millipore Corp. USA) were used in TBST with 3% BSA overnight at 4 °C. Dye 680 or 800 secondary antibodies were added at a concentration of 1:10,000 for 1 h at room temperature (LI-COR Biosciences, NE, USA). Detection was carried out with the LI COR Odyssey. Quantification of signals was carried out with the Odyssey program. The ratio between the intensity of the band of the tested protein and the intensity of the corresponding ACTIN or GAPDH band was calculated for normalization/expression of results.

### Nuclear cytoplasm extraction

Cardiac tissue (n = 4 in each group) was homogenized and the nuclear and cytoplasm fraction extracted using the NE-PER nuclear and cytoplasmic extraction kit (Thermo Scientific, IL, USA) according to the manufacturer’s instructions.

### Rt-PCR

Total RNA (n = 4 in each group) was purified from hearts using TRIzol (Ambion, Austin, TX, USA) as per manufacturer’s instructions. The quantity of total RNA was determined by OD260 measurements. cDNA was synthesized from total RNA using the TaqMan High Capacity cDNA Reverse Transcription Kit (Applied Biosystems, Foster City, CA, USA) according to the manufacturer’s protocol.

Quantitative real-time PCR analysis was performed using the step one plus system (Applied Biosystems, Foster City, CA, USA). The primers and TaqMan FAM probes were ordered from Applied Biosystems. All samples were normalized to an endogenous gene, mouse TATA-box.

**Table:** 

Gene	Assay ID
Tbp (TATA BOX)	Mm00446973
TNFα	Mm00443260
Tgfb	Mm01178820
Mmp2	Mm00439498
Nppa (ANP)	Mm01255747
Nppb (BNP)	Mm01255770
Ppargc1 (PGC-1α)	Mm01208835
Adipoq (adiponectin)	Mm00456425_

### Serum thiobarbituric acid reactive substances

Malondialdehyde (MDA) level is commonly known as a marker of oxidative stress. MDA can be quantified through a controlled reaction with thiobarbituric acid, generating ‘thiobarbituric acid reactive substances’ (TBARS). Thus, lipid peroxidation was determined (n = 4 in each group) using the TBARS assay kit (Cayman Chemical, MI, USA) according to the manufacturer’s instructions.

### SIRT and HDAC1/2 activity

SIRT and HDAC1/2 activity in the nuclear fraction of cardiac tissue samples (n = 4 in each group) were measured using an Universal SIRT activity assay kit (Abcam, Cambridge, UK) and a HDAC colorimetric assay/drug discovery kit (Enzo Life Sciences, NY, USA) according to the manufacturers’ instructions.

### Cell culture

Rat hearts (Sprague–Dawley, 1–2 days old) were removed under sterile conditions and washed three times in phosphate-buffered saline (PBS) to remove excess blood cells. The hearts were minced and then gently agitated in a solution of proteolytic enzymes—RDB (Biological Institute, Ness-Ziona, Israel), which was prepared from fig tree extract. RDB was diluted 1:100 in Ca^2+^ and Mg^2+^ free PBS for a few cycles of 10 min each, as described previously [35]. Dulbecco’s modified Eagle’s medium (Biological Industries, Kibbutz Beit Haemek, Israel) containing 10% horse serum was added to supernatant suspensions containing dissociated cells. The mixture was centrifuged at 300 g for 5 min. The supernatant was discarded, and the cells were resuspended. The suspension of the cells was diluted to 1.06 × 10^6^ cells/ml, and 1.5 ml of the suspension was placed in 35-mm plastic culture dishes, or 0.5 ml in 24 wells plates. The cultures were incubated in a humidified atmosphere of 5% CO2 and 95% air at 37^O^C. Confluent monolayers exhibiting spontaneous contractions developed in culture within 2 days.

### Experiments with EX-527

Cultured cardiomyocytes were incubated with normal (17.5 mM) or high concentration of glucose (33 mM) for 4 days. Glucose concentration of 17.5 mM is normally used for cardiomyocytes and was therefore considered as control. The SIRT 1 inhibitor EX-527 (10 µM) (Cayman Chemical, MI, USA) was added to the culture for 24 h.

### In-vitro ROS production measurement

ROS was detected using a 2′,7′-Dichlorofluorescin diacetate (DCF-DA) reagent (Sigma-Aldrich, St. Louis, MO,USA). This compound is an uncharged cell-permeable molecule. Inside cells, this probe is cleaved by nonspecific esterases, forming carboxydichlorofluoroscein, which is oxidized in the presence of ROS. Cells were loaded with 10 µM DCF-DA for 30 min at 37 °C and then washed. Fluorescence was monitored with a microplate fluorometer using wavelengths of 485/538 nm for excitation/emission, respectively.

### Statistical analysis

Animals were assigned to groups randomly. All values are expressed as mean ± SD. In the in vivo studies results were normalized to the WT group and in the in vitro studies the result were normalized to the 17.5 mM glucose control group. The statistical difference between the two groups was assessed using the 2-tailed student’s t test. To compare more than 2 groups, one way analysis of variance (ANOVA) with Duncan’s multiple comparison option was used. p < 0.05 was considered significant.

## Results

### Angiotensin II promotes development of metabolic dysfunction and diabetic cardiomyopathy

**Fig. 1:**
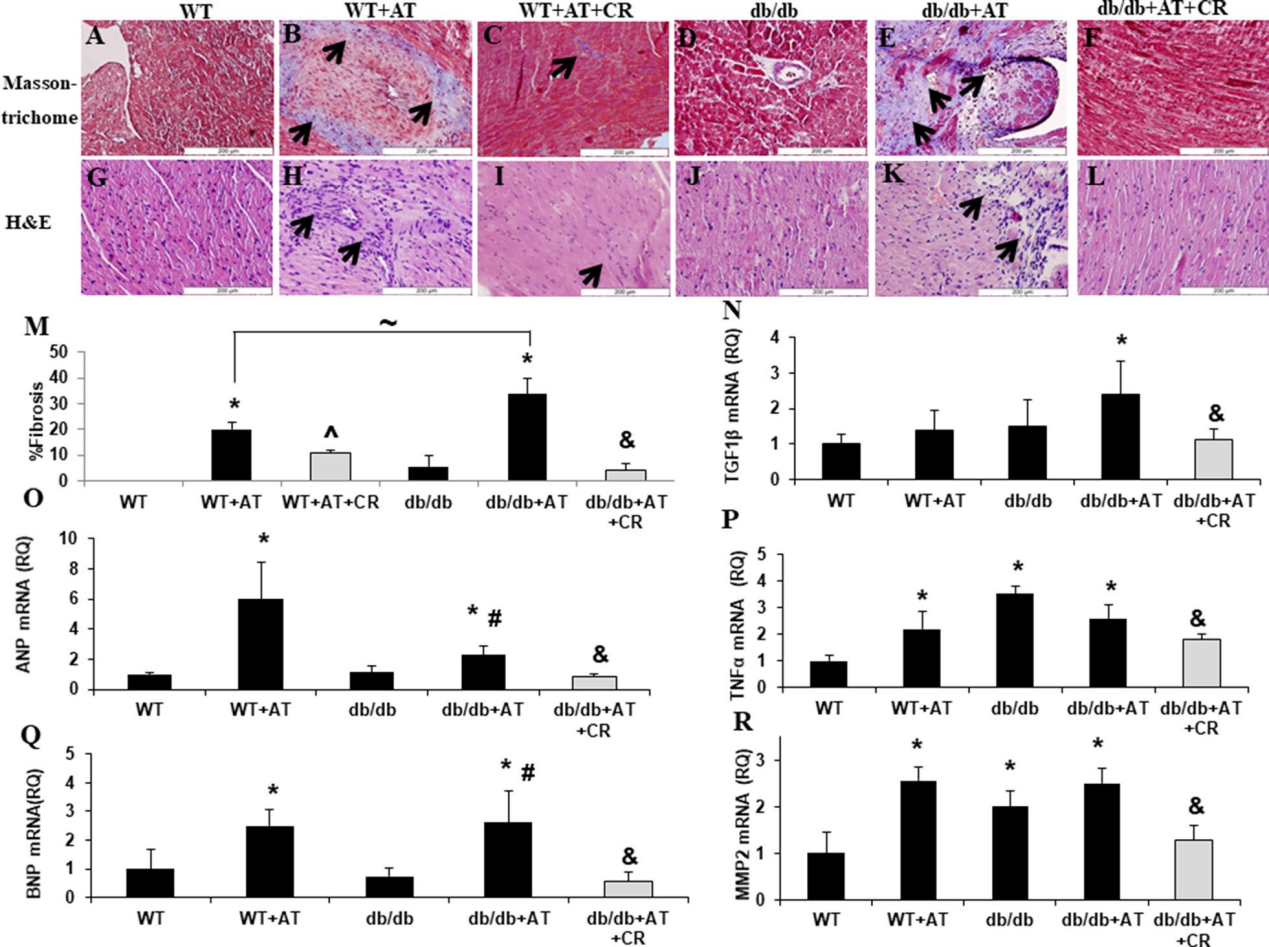
CR attenuates fibrosis and inflammation in diabetic mice. Representive pictures of leukocytes infiltration and fibrotic tissue (arrows) that were observed in the heart of AT treated mice as seen by Masson’s trichrome (**A**–**F**) and Hematoxylin &Eosin (**G**–**I**) staining. The percentage of fibrotic area was calculated (**M**). *p < 0.05 vs. WT, ^^^p < 0.05 vs. *WT *+ *AT,* ^&^p < 0.05 vs. *db/db *+ AT. n = 3 in each group. Values represent mean ± SD. CR reduced the cardiac mRNA levels of TGFβ (**N**), ANP (**O**), TNFα (**P**), BNP (**Q**), MMP2 (**R**). *p < 0.05 vs. WT, #p < 0.05 vs. db/db, &p < 0.05 vs. db/db + AT. n = 5 in each group. Values represent mean ± SD

DM had no significant effect on murine cardiac structure and function up to the age of 4 months. Diabetic mice (*db/db*) had normal cardiac function, dimension parameters and heart weight (Table 1) which was not significantly different from untreated WT mice. AT significantly (p < 0.05) increased blood pressure and heart weight in both WT and diabetic mice. AT treatment of *db/db* mice resulted in wall thickening and 10% reduction of LV diameter without change in systolic function (Table 1). In contrast, in WT mice, AT increased the LV dimension and impaired the systolic function without wall thickening (Table 1). AT had no effect on blood glucose levels but elevated the level of serum cholesterol and triglycerides in both WT and diabetic mice. Notably, blood lipids are already elevated in diabetic mice (Table 1). Histological staining demonstrated that AT promoted the formation of fibrotic tissue (p < 0.003) (Fig. 1A, B, D, E and M) and leukocyte infiltration (Fig. 1G, H, J, K). These pathological findings were more pronounced in the diabetic heart (p = 0.03, Fig. 1M).

**Table 1:** Metabolic and cardiac phenotype in AT treated mice

	WT	WT + AT	WT + AT + CR	db/db	*db/db *+ AT	*db/db *+ AT + CR
IVS; d (mm)	0.8 ± 0.1	0.9 ± 0.1	0.8 ± 0.2	0.9 ± 0.1	1.1 ± 0.1^#^	1 ± 0.1^&^
IVS; s (mm)	1.2 ± 0.1	1.2 ± 0.2	1.4 ± 0.5	1.4 ± 0.2*	1.6 ± 0.2^#^	1.5 ± 0.3*
LVPW; d (mm)	0.9 ± 0.1	1 ± 0.1	0.8 ± 0.1^**^**^	0.9 ± 0.1	1.1 ± 0.2^#^	0.9 ± 0.2^&^
LVPW; s (mm)	1.2 ± 0.1	1.1 ± 0.1	1.3 ± 0.1	1.2 ± 0.2	1.5 ± 0.3^#^	1.2 ± 0.3^&^
LVEDD (mm)	3.6 ± 0.7	4.2 ± 0.4*	3.5 ± 0.5^**^**^	3.9 ± 0.2	3.5 ± 0.05^#^	4.1 ± 0.4^&^
LVESD (mm)	2.9 ± 0.2	3.3 ± 0.6	1.93 ± 0.8^**^**^	2.6 ± 0.3*	2.4 ± 0.6*	2.5 ± 0.5
FS (%)	33 ± 14	7 ± 21*	14 ± 46^**^**^	34 ± 7	30 ± 7	41 ± 10^&^
Body weight (g)	26 ± 3	252±	191 ± *	41 ± 10*	40 ± 5*	33 ± 7^&^
Heart weight (mg)	115 ± 2	148 ± 3*	97 ± 2^**^**^	117 ± 2	163 ± 3^#^	139 ± 2^&^
Systolic blood pressure (mmHg)	95 ± 21	134 ± 29*	117 ± 13	99 ± 30	148 ± 15^#^	114 ± 11^&^
Glucose (mg/dL)	137 ± 44	111 ± 41	107 ± 43	617 ± 93*	658 ± 107*	531 ± 127^&^
AST (U/L)	62 ± 25	60 ± 11	60 ± 18	127 ± 53*	226 ± 149^#^	99 ± 21^&^
ALT (U/L)	126 ± 42	130 ± 30	127 ± 56	182 ± 134	281 ± 176*	117 ± 32^&^
Cholesterol (mg/dL)	79 ± 24	101 ± 22*	8 ± 70^**^**^	112 ± 21*	199 ± 91^#^	118 ± 25^&^
Triglycerides (mg/dL)	124 ± 57	125 ± 25	22 ± 82^**^**^	185 ± 66*	208 ± 75*	127 ± 35^&^

Values are mean±SD

*IVS;d* intra ventricular septum in diastole, *IVS;s* intra ventricular septum in systole, *LVPW;d* left ventricle posterior wall in diastole, *LVPW;s* left ventricle posterior wall in systole, *LVESD* left ventricle end systolic dimension, *LVEDD* left ventricle end diastolic dimension, *FS* fractional shortening, *AST* aspartate aminotransferase, *ALT* alanine aminotransferase

*p < 0.05 vs. WT, ^**^**^p<0.05 vs.WT+AT, #p<0.05 vs. *db/db*, &p<0.05 vs. *db/db*+ AT

### CR attenuates the metabolic abnormalities and development of diabetic cardiomyopathy

In both lean and diabetic mice, CR led to a ~ 20% weight loss. CR attenuated the AT-induced hypertension but this was statistically significant only in diabetic mice (p < 0.004). High glucose levels in the diabetic mice were only moderately reduced after CR (24%). Both in lean and in diabetic mice the increase in lipid profile following AT was attenuated (~ 65%) (Table 1). The left ventricular FS increased concomitant with attenuation of left ventricular hypertrophy (Table 1). Histopathological sections indicated an absence of fibrosis (p < 0.002, Fig. 1C, F and M) and leukocyte infiltration in cardiac tissue of the CR groups (Fig. 1I, L).

### Effect of CR on the molecular markers of cardiomyopathy

Genes related to cardiac hypertrophy and heart failure ANP, BNP were elevated in mice treated with AT independently of diabetes, and their expression was attenuated after CR (Fig. 1O and Q). Genes related to cardiac remodeling of TGFβ, TNFα and MMP2 were elevated by either diabetes or AT and were reduced in CR-mice (Fig. 1N, P and R).

### Effect of CR on metabolic signaling and SIRT activity

**Fig. 2:**
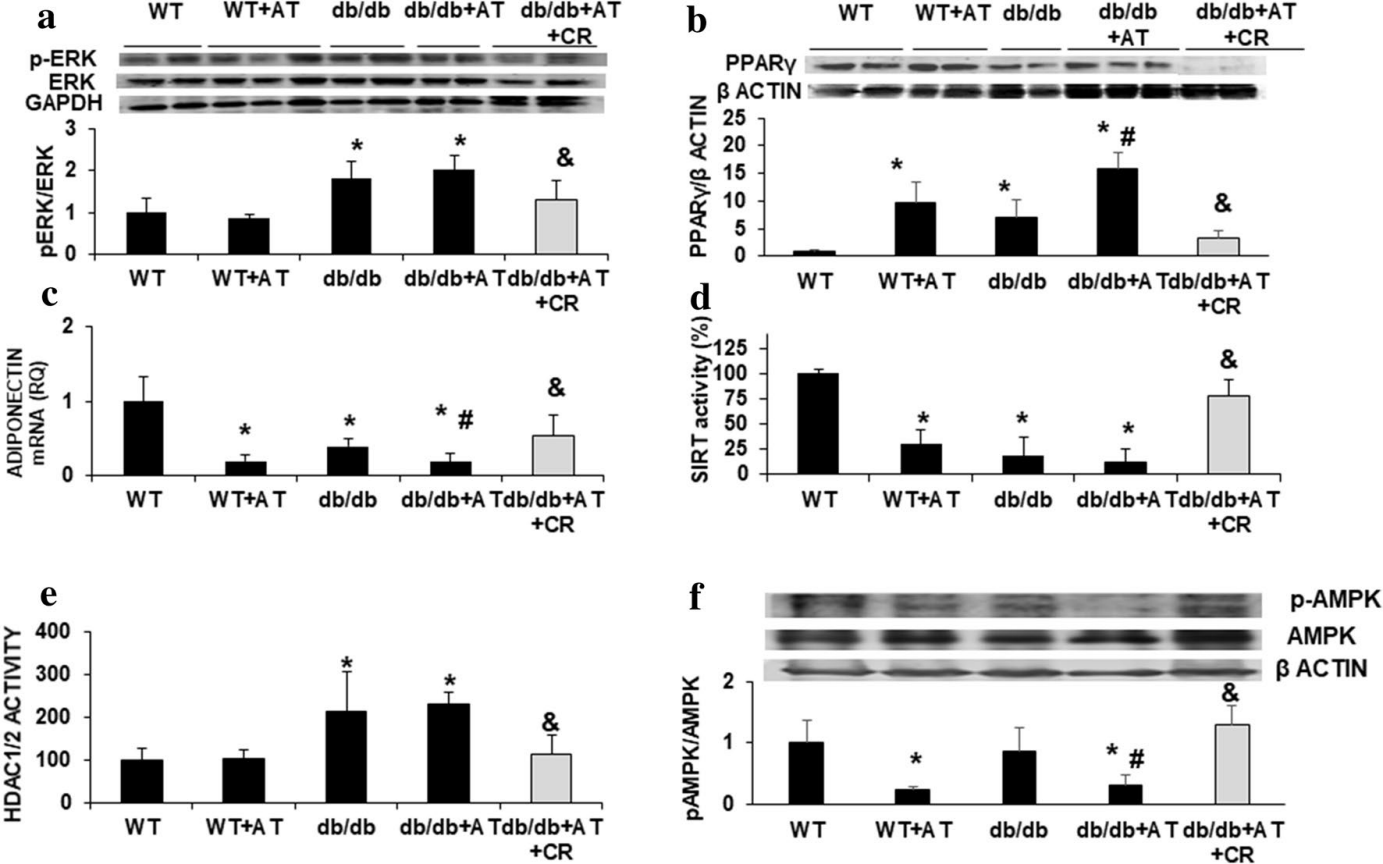
CR alters cellular metabolism and gene expression regulation. Representative Western blot for ERK1/2 phosphorylation and densitometry analysis of ERK1/2 normalized to GAPDH indicating attenuation in ERK overactivation following CR. n = 4 in each group, *p < 0.05 vs. WT, ^&^p < 0.02 vs. *db/db *+ AT group (**a**). Representative Western blot for PPARγ and densitometry analysis of PPARγ normalized to β actin showing reduction in PPARγ protein levels following CR. n = 4 in each group, *p < 0.03 vs. WT, ^#^p = 0.02 vs. *db/db*, ^&^p < 0.001 vs*. db/db *+ AT group (**b**). ADIPONECTIN mRNA levels were measured in the cardiac tissue (**c**). SIRT (**d**) HDAC1/2 (**e**) activity were measure in the nuclear fraction from WT and *db/db* mice hearts. Cardiac protein levels of pAMPK relative to total AMPK western blot and densitometry (**f**). *p < 0.05 vs. WT, ^#^p < 0.05 vs. *db/db*, ^&^p < 0.05 vs*. db/db *+ AT group, n = 4 in each group. Values represent mean ± SD

Phosphorylation of ERK1/2 is part of the stress signaling in the heart. ERK1/2 was activated in diabetic mice independently of AT infusion. Its phosphorylation increased by twofold (p < 0.02) but was reduced by 70% (p < 0.05) due to CR (Fig. 2a). The cardiac expression of adiponectin was reduced by AT in both WT and diabetic mice. CR increased the expression of adiponectin by threefold (p < 0.04), (Fig. 2c). pAMPK was reduced in the AT-treated mice (p < 0.04) and was elevated following CR (p = 0.001) (Fig. 2f). In diabetic mice, PPARγ levels were sevenfold higher compared to WT mice and were further elevated after AT treatment. CR reduced PPARγ levels (fivefold reduction) in the heart of *db/db* +AT mice indicating its modifying effect on cellular metabolism (Fig. 2b).

In WT mice, SIRT activity was reduced by 70% after AT treatment (p = 0.003). In *db/db* and *db/db *+ AT mice, SIRT activity was reduced by 82 and 88% respectively compared to WT mice (p = 0.02). CR increased SIRT activity in *db/db *+ AT mice by 65% (p < 0.05) (Fig. 2d). SIRT1 can affect chromatin remodeling and thus modulation of gene expression [36]. The suppression of SIRT activity in the diabetic mice resulted in ~ twofold (p < 0.04) elevation in HDAC 1/2 (histone deacetylase) activity that was prevented by CR (p = 0.002), (Fig. 2e).

### CR reduced oxidative stress and increased levels of PGC-1α

**Fig. 3:**
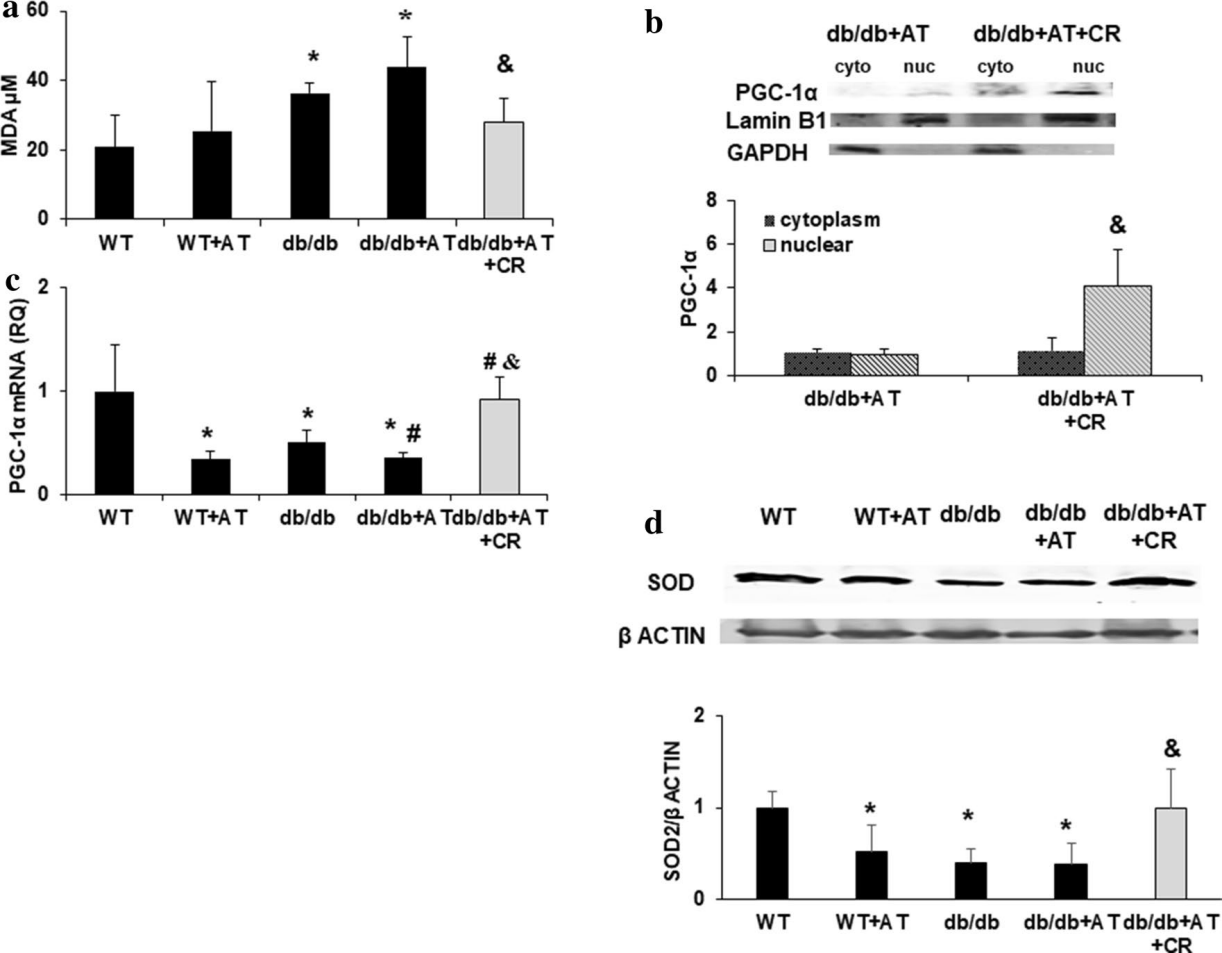
CR alleviates oxidative stress through the activation of PGC-1α. MDA levels in the serum were measured using TBARS kit, n = 4 in each group (**a**). PGC-1α protein levels in the cytoplasmic (cyto) and nuclear (nuc) fractions (**b**) as well as mRNA expression levels (**c**) were measured in the cardiac tissue (**c**). Cardiac SOD2 protein levels (**d**). n = 4 in each group, *p < 0.05 vs. WT, #p < 0.05 vs. *db/db* &p < 0.05 vs. *db/db *+ AT. Values represent mean ± SD

In order to clarify the effect of CR on oxidative stress and anti-oxidative defense, we measured MDA levels in the serum and found them to be significantly elevated in *db/db *+ AT mice compared to WT mice (p = 0.01). CR reduced the levels of MDA in the serum (p < 0.03) (Fig. 3a). SIRT1 regulates PGC-1α thus activating anti-oxidant defenses [37]. The level of mitochondrial factor PGC-1α mRNA levels were reduced by either AT or diabetes (p < 0.001). Following CR, PGC-1α levels were elevated (p < 0.0001) and were restored to WT levels (Fig. 3c). The PGC-1α protein levels were increased in the nuclear but not cytosolic fraction of CR-treated diabetic mice (Fig. 3b). These changes concurred with the level of antioxidant defenses represented by SOD2 protein levels (Fig. 3d).

### SIRT1 controls PGC-1α levels and is involved in cellular ROS levels

**Fig. 4:**
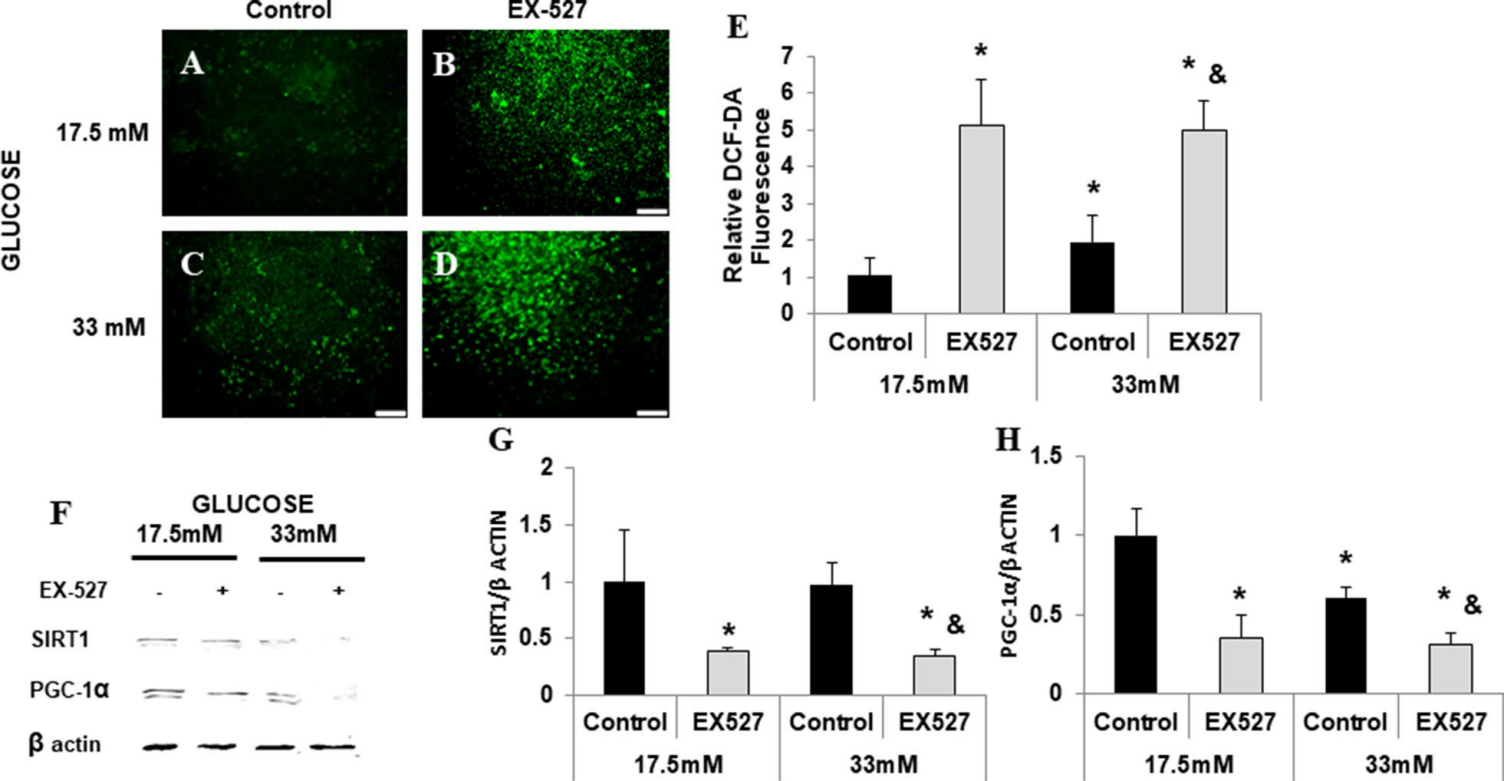
SIRT1 is required for the expression of PGC-1α. Neonatal Rat cardiomyocytes were exposed to 17.5 and 33 mM glucose and treated with the SIRT1 inhibitor EX-527. Cells were stained with DCF-DA (**A**–**D**) and fluorescence was measured using fluorimeter (**E**). Representative western blots for SIRT1 and PGC-1α for cell treated with EX-527 (**F**). Densitometry analysis for SIRT1 (**G**) and PGC-1α (**H**). Results were normalized to the group exposed to 17.5 mM glucose. *p < 0.04 vs.17.5 mM control, ^&^p < 0.02 vs. 33 mM control, n = 4 in each group. Values represent mean ± SD

**Fig. 5:**
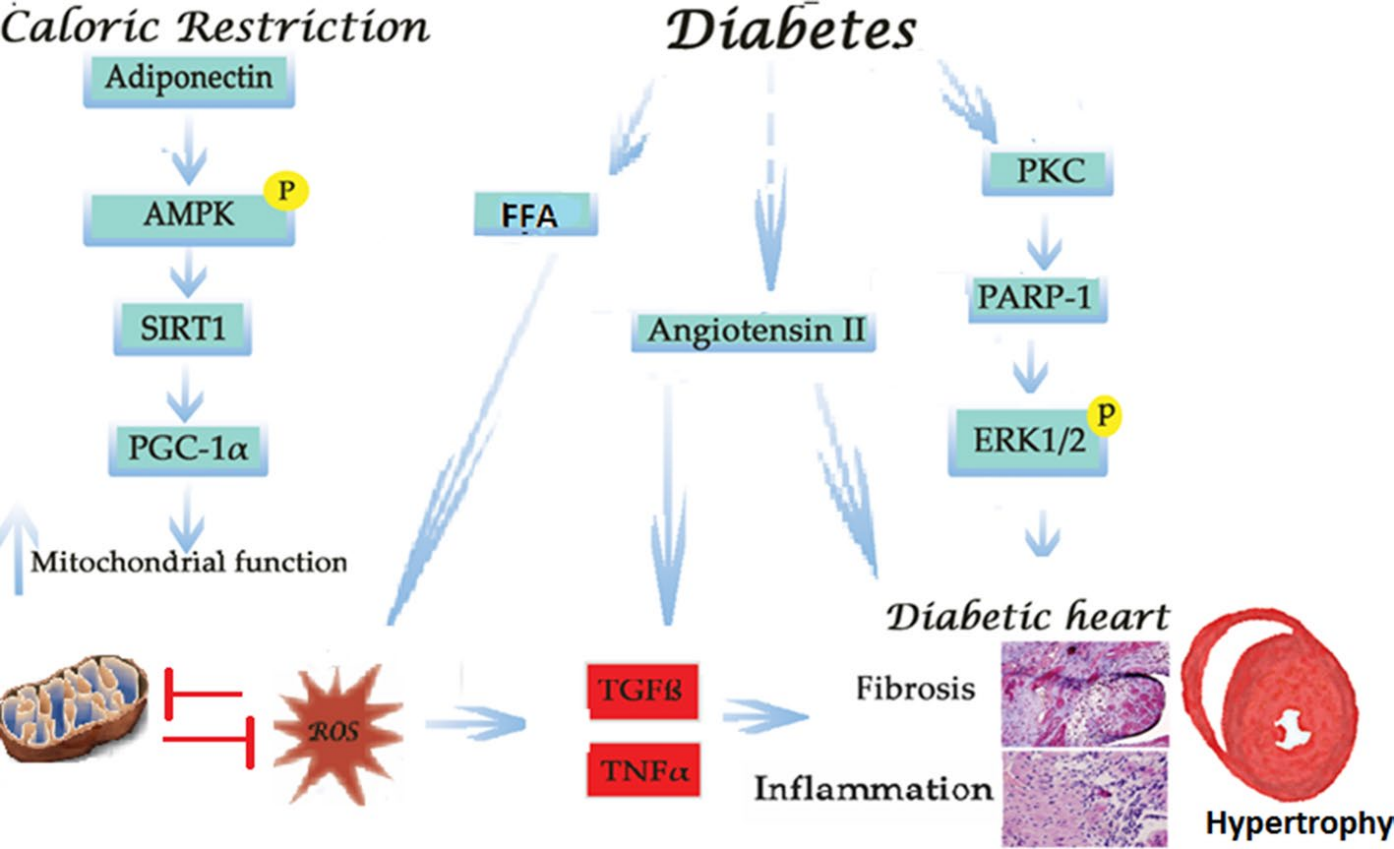
Schematic description of the cellular signaling involved in the development of cardiomyopathy in diabetes and proposed mechanism for the effect of CR on the diabetic heart. The energetic dysfunction in diabetes manifested by increased FFA in the heart together with elevation in local and systematic production of Angiotensin leads to mitochondrial dysfunction, oxidative stress and inflammation. In the diabetic heart PARP-1 and ERK are elevated, promoting the development of cardiac hypertrophy. CR elevates adiponectin, pAMPKand SIRT-1 levels. This leads to the activation of both PGC-1α and improved mitochondrial function, alleviate the oxidative stress and reduce inflammation by CR ameliorating cardiomyopathy

The relationships between SIRT1 and PGC-1α in response to hyperglycemic were tested in vitro. To examine the role of SIRT1 in glucose metabolism and oxidative stress we used cultured rat neonatal cardiomyocytes exposed to high concentration of glucose (33 mM). Elevation of glucose levels led to increased cellular ROS production (p < 0.03) (Fig. 4A and C) and reduction in PGC-1α protein levels (p < 0.05, Fig. 4F and H). SIRT 1 inhibition by EX-527 elevated ROS production (Fig. 4B and D). SIRT1 and PGC-1α levels were dramatically reduced (Fig. 5f–h). Cumulatively these results indicate the pivotal role and the direct relationship between SIRT1 and PGC-1α protecting cardiomyocytes against oxidative stress which participates in the pathogenesis of diabetic heart disease.

### Discussion

In the normal heart, insulin stimulates glucose uptake and oxidation. Insulin binding to its surface receptor elicits a signaling cascade that includes activation of PI3 K, AKT and PKC leading to the translocation of glucose transporters to the cardiomyocyte membrane thus facilitating glucose uptake. Insulin resistance in diabetic patients results in impaired cardiac glucose uptake and hyperglycemia, whereas cardiac FA uptake and metabolism increase [38]. This initial switch towards FA utilization elevates intracellular FA derivatives, such as MDA, which activate the PPARα/γ. Subsequently, there is an increase in ROS generation and oxidative damage in the mitochondria isolated from diabetic hearts. Diabetes is characterized by a decreased expression of oxidative phosphorylation genes. Many of these genes are regulated by nuclear respiratory factor 2 (NRF2)-dependent transcription of antioxidant enzymes, such as superoxide dismutase (SOD), catalase, Heme Oxygenase -1 (HO-1), NAD (P) H, quinine oxidoreductase-1 and thioredoxin (Trx-1). When antioxidant defenses are insufficient to counteract ROS production, the excessive levels of ROS become cytotoxic [39]. Diabetic hearts are characterized by reduced AKT phosphorylation [40] and by decreased mitochondrial function [41] resulting in the overwhelming of the endogenous protective mechanisms and defective responsiveness to stress.

In this study we examined the ability of CR to alleviate diabetic cardiomyopathy and to identify the key pathways involved in CR mediated cardioprotection. CR is consider to be metabolic protective, however there are conflicting report as to its effects on the heart in diabetes [42]. There are numerous pathways involved in different models of cardiomyopathy [43]. The renin angiotensin system (RAS) plays a fundamental role in the pathophysiology of diabetes and its complications, as a result angiotensin-converting enzyme (ACE) inhibitors are widely used in treating diabetic patients [44]. Cardiac hypertrophy is mediated in part by RAS and TGF-β which have a central role in cardiac remodeling. Diabetic mice 4 months old did not develop cardiac hypertrophy or fibrosis. Therefore, we used AT in our model system to enhance and aggravate cardiomyopathy that otherwise only occurs in obese diabetic mice with aging. This concentration was reported previously to induce cardiomyopathy mainly through its profibrotic effects [2, 12]. AT through AT1R stimulates TGF-β which promotes fibroblast proliferation, ECM deposition and myocyte hypertrophy [45] independent of its effects on blood pressure [12]. TGF-β induction precedes the development of myocardial fibrosis and ECM production [46]. Noteworthy, ERK1/2 and TGF-β were elevated in the diabetic mouse independent of AT, indicative of cellular signaling that precedes the development of cardiomyopathy. In addition to its effects on myocardial remodeling AT contributed to the dyslipidemia in *db/db* mice and increased PPARγ expression. These changes may underlie additional detrimental effects such as increased lipid accumulation and atherosclerosis [47], which are often observed in diabetic patients.

CR reduced body weight and prevented the development of left ventricle hypertrophy. The cardioprotection afforded by CR was unrelated to glucose levels which were only mildly reduced. A possible explanation is that the leptin signaling, which is abolished in *db/db* mice, is important in glucose homeostasis that is independent of the pathways by which leptin regulates food intake and body weight [48]. Furthermore, this proves that CR has a direct effect on cardiomyocytes beyond glycemic control. PPARγ is a regulator of lipid utilization and PPARγ agonists are used clinically as antidiabetic drugs [49]. However, a PPARγ activator, like thiazolidinediones, is associated with side effects such as edema and body weight gain [50, 51]. It is suggested that using a PPARδ specific ligand can be a more beneficial therapeutic approach to treat the diabetic heart [52]. CR reduced cardiac PPARγ levels, concomitantly attenuating cardiac lipotoxicity which is one of the mechanisms responsible for diabetic cardiomyopathy and heart failure [53].

In isolated cardiomyocytes AT increased ERK1/2 phosphorylation in a Ca^+^ dependent manner, which led to the upregulation of ANP [54]. These effects may be direct, via activation of ERK-1/ERK-2-dependent pathway, increasing the local production of TGF-β [55]. Cardiac hypertrophy is associated with alterations in gene expression involving epigenetic changes [56]. The development of cardiac hypertrophy in our mouse model was associated with increased HDAC activity and ERK phosphorylation. It was attenuated by CR (Fig. 2).

Sirtuins are NAD+ -dependent, and, as such, their activation is regulated by the cellular metabolic state [57]. One of the key metabolic pathways is activation of sirtuin 1 (SIRT1) resulting in increased levels of adiponectin and stimulation of mitochondrial biogenesis and antioxidant defenses. SIRT1 regulates many essential cellular processes including survival, apoptosis, inflammation, stress resistance, cell growth, metabolism and senescence and prevent mitochondrial dysfunction [58, 59]. The expression of SIRT1 was shown to be reduced in a mouse model of diabetic cardiomyopathy and resulted in both compromised insulin signaling and mitochondrial dynamic abnormity. Whereas deletion of SIRT1 expression in the heart contributed to phenotypes resembling diabetic cardiomyopathy in vivo and to mitochondrial dysfunction through PGC1-α in vitro [60, 61]. Thus Sirt1 activators such as resveratrol can used as CR mimetics to reduce diabetic cardiomyopathy.

Several signaling pathways converge at the level of the mitochondria therefore accounting for cardiac mitochondrial dysfunction in diabetes. AMPK is a key metabolic regulator activated during exercise and weight loss. Among the many activities of AMPK are transcription and phosphorylation of the co-activator PGC-1α, which induces mitochondrial transcription factors (e.g. mtTFA) augmenting both mitochondrial content and cellular metabolic oxidative capacity. Yet, PGC-1α is not activated until it is deacetylated by SIRT1 [62, 63]. Dysfunction in this pathway has been implicated as a contributing factor in such metabolic disorders as the metabolic syndrome and DM2 [64]. Additionally, there is a direct relationship between SIRT1 and NO production and vascular function in DM2 patients [65]. Oxidative stress plays a pivotal role in the development of obesity and the pathogenesis of diabetes [8]. PGC-1α is a master regulator of mitochondrial biogenesis. PGC-1α is activated by both SIRT1 through deacetylation and by AMPK through phosphorylation, resulting in improved mitochondrial function [26, 27]. Mitochondrial malfunction associated with insulin resistance is a prime contributing factor for DCM. Forkhead box transcription factor 1 (FOXO1) is involved in the integration of mitochondrial function with insulin signalling. Elevated FOXO1 levels in insulin resistant states disrupt mitochondrial electron transport chain, thereby promoting impaired oxidative respiration. In addition, FOXO1 is involved in the regulation of the mitochondrial biogenesis by affecting the expression of genes regulating mitochondrial fission and fusion through SIRT1/PGC-1α pathway [66]. Another novel factor regulation PGC-1α activity is Extracellular signal-regulated protein kinase 5 (Erk5) which is lost in the hearts of obese/diabetic animal models [41].

We have previously shown that activation of PGC-1α reduced mitochondrial ROS and prevented adipogenesis in adipocytes [67]. EX-527 mediated inhibition of SIRT1 elevated ROS production while SIRT1 and PGC-1α levels were dramatically. In higher glycemia, PGC1 α inhibition induced increased mitochondrial fragmentation and ROS generation [68]. In this study we show that the cardioprotective effect of CR in diabetic mice involves increased expression of PGC-1α and SIRT1. In cardiomyocytes, pharmacological inhibition of SIRT1 was followed by the decreased expression of PGC-1α and increased ROS production. We conclude that the increased expression of SIRT1 and PGC-1α is central to rebuffing oxidative stress caused by hyperglycemia and thus a therapeutic approach to cardiac protection.

We have previously shown that activation of PGC-1α reduced mitochondrial ROS in adipocytes through the induction of HO-1 [67] and its downstream targets constituting essential antioxidant defense mechanisms. HO-1 has been directly implicated in the protection of the diabetic heart [69, 70]. Silencing PGC-1α by the use of antisense nucleotides prevented increased levels of HO-1 [67]. We conclude that the SIRT1-PGC-1α-HO-1 axis is pivotal in order to cope with oxidative stress caused by hyperglycemia and has an important role to play in protecting the diabetic heart.

While the field of pharmacological therapies continues to expand, efforts to facilitate weight loss have had limited success. In the present study we examined the cellular mechanism by which CR protects the diabetic heart. A new class of drugs that mimic the cellular and metabolic of exercise or calorie restriction would lead to, we believe, better patient compliance than a true calorie restriction.

Understanding the fundamental and biochemical processes to prevent adverse cardiac remodeling that occur in diabetic cardiomyopathy is crucial for developing novel therapeutic approaches to treat heart failure, a major source of morbidity and mortality in diabetic patients and a major drain of health care costs and resources.
