# Correction to: Regulation of diabetic cardiomyopathy by caloric restriction is mediated by intracellular signaling pathways involving ‘SIRT1 and PGC-1α’

**DOI:** 10.1186/s12933-018-0757-1

**Published:** 2018-08-17

**Authors:** Maayan Waldman, Keren Cohen, Dor Yadin, Vadim Nudelman, Dan Gorfil, Michal Laniado-Schwartzman, Ran Kornwoski, Dan Aravot, Nader G. Abraham, Michael Arad, Edith Hochhauser

**Affiliations:** 1 Cardiac Research Laboratory, Felsenstein Medical Research Institute Petah-Tikva, Sackler Faculty of Medicine, Tel Aviv University, Tel Aviv, Israel; 2 Leviev Heart Center, Sheba Medical Center, Tel Hashomer and Sackler School of Medicine, Tel Aviv University, Tel Aviv, Israel; 3 Department of Pharmacology, New York Medical College, Valhalla, NY 10595 USA; 4 Felsenstein Research Center, Rabin Medical Center, Sackler Faculty of Medicine, Tel Aviv University, Jabotinsky St, 49100 Petach Tikva, Israel

## Correction to: Cardiovasc Diabetol (2018) 17:111 10.1186/s12933-018-0754-4

Unfortunately, after publication of this article [1], it was noticed that Table 1 contained errors introduced during the production process. In the WT + AT column, the value for the FS row is 21 ± 7 and the value for the Body Weight row is 25 ± 2. In the WT + AT + CR column, the value for the FS row is 46 ± 14 and the value for the Body Weight row is 19 ± 1. The original article has been corrected.
